# Impact of discrepancies between CT numbers of brain-tissue-equivalent density plug and actual brain tissue on dose calculation accuracy

**DOI:** 10.1007/s12194-025-00908-z

**Published:** 2025-05-12

**Authors:** Shogo Tsunemine, Shuichi Ozawa, Minoru Nakao, Satoru Sugimoto, Tetsuya Tomida, Michitoshi Ito, Masumi Numano, Hideyuki Harada

**Affiliations:** 1 Department of Radiation and Proton Therapy Office, Shizuoka Cancer Center, Shizuoka, Japan; 2 Hiroshima High-Precision Radiotherapy Cancer Center, Hiroshima, Japan; 3 Department of Radiation Oncology, Graduate School of Biomedical & Health Sciences, Hiroshima University, Hiroshima, Japan; 4 Medical Data Mathematical Reasoning Team, Advanced Data Science Project, Information R&D and Strategy Headquarters, RIKEN, Yokohama, Kanagawa Japan; 5 Department of Radiation and Proton Therapy Center, Shizuoka Cancer Center, Shizuoka, Japan

**Keywords:** Brain-tissue-equivalent density plug, CT-RED/MD, Inhomogeneity correction

## Abstract

This study quantitatively evaluated the impact of differences in computed tomography (CT) numbers and elemental compositions between commercially available brain-tissue-equivalent density plugs (BDPs) and actual brain tissue on dose calculations in a radiation therapy treatment planning system (RTPS). The mass density and elemental composition of BDP were analyzed using elemental analysis and X-ray fluorescence spectroscopy. The CT numbers of the BDP and actual brain tissue were measured and compared, with effective atomic numbers (EANs) calculated based on compositional analysis and the International Commission on Radiological Protection Publication 110 data for brain tissues. The theoretical CT numbers were derived using the stoichiometric CT number calibration (SCC) method. The dose calculations were performed using the modified CT number-to-relative electron density (RED) and mass density (MD) conversion tables in Eclipse v16.1, employing AAA and Acuros XB algorithms, employing the physical material table in AcurosXB_13.5. The dose metrics D_2%_, D_50%_, and D_98%_ were evaluated. Significant differences in elemental composition were found, particularly in carbon (73.26% in BDP vs. 14.3% in brain tissue) and oxygen (12.52% in BDP vs. 71.3% in brain tissue). The EANs were 6.6 for BDP and 7.4 for brain tissue. The mean CT numbers were 23.30 HU for the BDP and 37.30 HU for brain tissue, a 14 HU discrepancy. Nevertheless, dose calculation deviations were minimal, typically within ± 0.2%, with a maximum discrepancy of 0.6% for D_98%_. Although CT numbers and elemental compositions exhibited notable differences, their impact on dose calculations in the evaluated RTPS algorithms was negligible.

## Introduction

Improving the accuracy of dose calculations in radiation therapy is essential for precisely evaluating the treatment efficacy and minimizing potential adverse effects. A critical component in achieving accurate dose calculations involves using a computed tomography (CT) number-to-relative electron density (RED) and mass density (MD) conversion table (CT-RED/MD). This table is particularly important for implementing CT-based inhomogeneity corrections to ensure variations in tissue composition and density are appropriately accounted for during the treatment planning process [1–4].

The CT-RED/MD table was user-defined and integrated into a radiation treatment planning system (RTPS). The influence of the accuracy of the CT-RED/MD table on dose distribution has been highlighted in several studies [5–9]. Several studies have established guidelines and tolerance levels for acceptable values in the CT-RED/MD tables. These guidelines ensure accurate dose calculations in radiation therapy treatment planning and minimize the uncertainties caused by variability in CT numbers and tissue characterization [8, 10, 11]. The calibration of the CT-RED/MD table using commercially available tissue-equivalent density plugs is a widely adopted approach in photon beam therapy. This method involves CT scanning of plugs with various densities to establish the relationship between the CT numbers and their corresponding known RED and MD values. Although this approach closely simulates clinical conditions, its accuracy depends on the plugs possessing RED and MD values that precisely represent real tissues.

In contrast, dose calculations in proton therapy use stoichiometric CT number calibration (SCC) to estimate the stopping power ratio. Schneider et al. reported that SCC achieved higher dose accuracy in Monte Carlo simulations than the plug-based calibration method [12]. The SCC establishes the relationship between CT numbers and stopping power ratios through theoretical calculations, thus eliminating the need for direct measurements using tissue-equivalent materials. This approach enhances accuracy by passing the potential discrepancies associated with material substitutes in conventional calibration methods [12, 13].

Our preliminary investigation revealed a significant discrepancy between the CT numbers of commercially available brain-tissue-equivalent density plugs (BDPs), the theoretical CT numbers calculated using the SCC method based on the International Commission on Radiological Protection (ICRP) Publication 110, and the actual CT numbers observed in actual brain tissue [14, 15]. While the issue with the brain plug became evident, it has also been shown that, for other tissues, the CT numbers of commercially available density plugs exhibit only minor discrepancies compared to values calculated from ICRU-referenced composition and density [16].

This discrepancy may affect the accuracy of dose calculations in RTPSs for brain cases. To address this issue, we conducted a comprehensive investigation that included a compositional analysis of commercially available BDP, a comparison of CT numbers between BDP and actual brain tissue, theoretical CT number calculations using the stoichiometric CT number calibration (SCC) method, the development of a CT number-to-relative electron density and mass density (CT-RED/MD) table based on the compositional analysis of BDP data, and an evaluation of the impact of the CT-RED/MD table on dose calculation accuracy within a commercial RTPS. This study aims to quantitatively assess the effects of discrepancies in CT numbers between BDP and actual brain tissue on dose calculation accuracy.

## Materials and methods

### Compositional analysis

We used Brain SR-2 (Gammex Inc., Middleton, Wisconsin, USA) as the BDP. To construct the SCC model, we analyzed the physical density and elemental composition of Brain SR-2 as well as previously analyzed lung-equivalent material (tough lung) and bone-equivalent material (Tough Bone) (Kyoto Kagaku Co., Ltd., Japan) [16]. For the compositional analysis of hydrogen (H), carbon (C), and nitrogen (N), we employed a 2400 II CHNS/O elemental analyzer (PerkinElmer Inc., Waltham, Massachusetts, USA). X-ray fluorescence spectroscopy was employed for the semi-quantitative analysis of elements, including aluminum (Al), silicon (Si), phosphorus (P), sulfur (S), chlorine (Cl), calcium (Ca), titanium (Ti), and barium (Ba).

### CT Number measurement of brain-tissue equivalent density plugs

CT scans were performed using a Gammex RMI 467 calibration phantom (Gammex Inc., Middleton, Wisconsin, USA) with various tissue-equivalent materials, including brain SR-2, tough lung, and tough bone, to obtain the actual CT numbers of the BDP. The scans were conducted using an Aquilion Prime Beyond CT scanner (Canon Medical Systems Corporation, Japan) under the following parameters: tube voltage: 120 kV; tube current: 300 mA; slice thickness: 2 mm; field of view (FOV): 500 mm; reconstruction kernel: FC03. At our institution, the FC03 reconstruction kernel is commonly applied in chest and abdominal imaging to ensure stable CT number measurements for homogeneous standard materials. Therefore, for the BDP measurements, we used FC03 to align with the CT-RED/MD table used in CT-RED/MD protocol. These conditions match those used to construct the CT-RED/MD table. For CT number measurements of each tissue-equivalent material, a circular region of interest (ROI) with a diameter of 1.53 cm was positioned at the center of each material slice. The CT numbers for all pixels within the ROI were recorded, with the mean CT number calculated as the average of all pixel values. The standard deviation (SD) within the ROI was also computed from a single measurement to evaluate the variability.

### CT number measurement and analysis of actual brain using clinical data

We analyzed CT data from ten patients who had previously undergone whole-brain irradiation for brain lesions to obtain CT numbers for actual brain tissue. The patients were randomly selected, with the study protocol approved by our institution’s ethics review board. CT scans were performed to measure the attenuation properties of brain tissue under the following conditions: tube voltage: 120 kV; tube current: 300 mA; slice thickness: 2 mm; FOV: 400 mm; reconstruction kernel: FC26. For brain tissue measurements, we used FC26, a high-contrast reconstruction kernel frequently employed in clinical brain imaging. This selection was made to better replicate actual clinical conditions, ensuring that the measured CT numbers accurately reflect those obtained in patient imaging. The same CT scanner described in Sect. 2.2 was used for all scans. The brain parenchymal regions were extracted using the auto-contouring function in Eclipse ver. 16.1 (Varian Medical Systems). The “actual brain” was defined as the whole-brain parenchyma excluding the gross tumor volume (GTV). The mean CT numbers and SDs were calculated for these regions. We first performed an overall comparison of all 10 cases and then conducted a detailed CT number histogram analysis for one representative case to illustrate the differences more clearly. The histogram was generated using CT number measurements from a circular region of interest (ROI) with a diameter of 1.53 cm, positioned at the center of each material slice. For the final analysis, the overall mean CT number and standard deviation (SD) were calculated as the average of all 10 cases.

### Comparison of effective atomic number

ICRP Publication 110 does not differentiate between white and gray matter in brain tissue, treating them as a single material despite their distinct compositions in practice. To maintain consistency with this reference data, our analysis focused on whole-brain tissue rather than separately analyzing white and gray matter.

The effective atomic numbers (EANs) of BDP were calculated using the following formula based on elemental composition analysis and data from ICRP Publication 110:

1 $${Z}_{\text{eff}}={\left(\frac{{\sum }_{i}\frac{{{\omega }_{i}Z}_{i}}{{A}_{i}}{Z}_{i}^{m}}{{\sum }_{i}\frac{{\upomega }_{i}{Z}_{i}}{{A}_{i}}}\right)}^\frac{1}{m}$$

*ω*_i​_ represents the mass fraction of the constituent elements *i*, while *Z*_i_ and *A*_i​_ denote the atomic number and atomic mass of each element, respectively. The exponent m, set to 3.3, reflects the balance between Compton scattering and the photoelectric effect within the effective energy range used in this study (50–60 keV). This choice is consistent with ICRU Report 46 data and shows excellent agreement with the reference ${Z}_{\text{eff}}$ values [17].

### Theoretical CT number calculation for brain density plug (BDP) and actual brain tissue using stoichiometric CT number calibration model

Theoretical CT numbers were calculated using the SCC method based on a previously established model [11]. The initial model parameters for the SCC method were determined using the least squares method and the Nelder–Mead simplex algorithm implemented in Python (https://docs.scipy.org/doc/scipy/reference/generated/scipy.optimize.fmin.html).

These model parameters, optimized to reproduce the CT numbers of the tough lung, tough bone, and air, were input into the Gammex RMI 467 phantom. We calculated the theoretical CT numbers for both the BDP and actual brain tissue using the SCC method with optimized model parameters. The compositional analysis results of the brain tissue-equivalent density plug obtained in Sect. 2.1 and the physical density and elemental composition data for actual brain tissue derived from ICRP 110 were adopted in the SCC method [14].

### Impact of CT-RED/MD conversion table modifications on dose calculations in brain cases

We performed dose calculations using modified CT-RED/MD tables to quantitatively assess the impact of CT number differences between the BDP and actual brain tissue on dose calculations. The calculations were conducted using Eclipse version 16.1, comparing the following three CT-RED/MD tables (Table 1):

1. The standard CT-RED/MD table currently used in clinical practice, which includes a brain tissue-equivalent density plug (BDP) as a reference (Reference).
2. A modified table excluding the BDP data points to assess how the absence of this density plug affects dose calculations (Modified (excluded BDP)).
3. A modified table utilizing the CT number of actual brain tissue, derived from ICRP Publication 110 data, to evaluate the effect of using a more physiologically accurate representation of brain tissue (Modified (ICRP110 Brain)).

**Table 1:** Comparison of CT-RED/MD tables: original and modified versions

MD (g/cm^3^)	RED	Original CT number (HU)	Modified (except BPD) (HU)	Modified (ICRP110 Brain) (HU)
0.001	0.001	− 1000	− 1000	− 1000
0.29	0.28	− 703	− 703	− 703
0.46	0.445	− 528	− 528	− 528
0.943	0.926	− 102	− 102	− 102
0.982	0.959	− 42	− 42	− 42
1	1	0	0	0
1.018		2	2	2
1.054	1.05	26		46
1.089	1.058	71	71	71
1.141	1.094	216	216	216
1.147	1.099	228	228	228
1.332	1.277	427	427	427
1.56	1.47	777	777	777
1.823	1.695	1228	1228	1228
5	5	4000	4000	4000

The “Original” column shows the standard CT-RED/MD table currently used in clinical practice. The “Modified (except BDP)” column shows the CT-RED/MD table with BDP data removed. The “Modified (ICRP 110 Brain)” column shows the CT-RED/MD table using ICRP 110 reference brain data

*MD* mass density, *RED* relative electron density, *BPD* brain-tissue-equivalent density plug

This comparison was designed to determine whether the presence of the BDP in the standard CT-RED/MD table introduces deviations in dose calculations and whether replacing it with a more accurate CT number from ICRP data would yield significant changes. By including these three conditions, we aimed to clarify how CT number differences in brain tissue modeling influence dose calculations in clinical settings.

For the dose calculations, we analyzed cases involving whole-brain irradiation (WBI) using a non-opposing four-field technique and brain stereotactic radiotherapy [SRS/SRT (stereotactic radiosurgery/stereotactic radiotherapy)]. The reason for selecting brain-related cases is to investigate the impact of the BDP on dose calculations. Furthermore, WBI and SRS/SRT were chosen to compare the effects on target volumes of different sizes, with WBI representing a large planning target volumes (PTV) and SRS/SRT representing a small PTV. The treatment plans for both SRS and SRT were generated using the dynamic conformal arc technique. The PTV for the three SRS and SRT cases were 7.13 cc, 11.27 cc, and 7.22 cc for Case 1, Case 2, and Case 3, respectively.

The dose calculation algorithms used were the anisotropic analytical algorithm (AAA) and Acuros XB (version 16.1.2), with a calculation grid size of 2.5 mm utilizing the physical material table in AcurosXB_13.5.We evaluated the dose–volume histogram (DVH) parameters D_2%_, D_50%_, and D_98%_. Here, Dx% represents the dose received by x% of the target volume. The Monitor Unit remained the same, with only the CT-RED/MD being changed for recalculation. All other parameters, including gantry angle, multi-leaf collimator positions, collimator angle, and jaw settings, were kept unchanged.

Using the dose calculation results obtained from the clinically utilized CT-RED/MD table as a reference, the relative dose differences between the reference and the other two tables were calculated using the following equation:

2 $$\Delta D= \frac{{D}_{mod}- {D}_{ref}}{{D}_{ref}} \times 100 \left(\%\right)$$

where *D*_ref_ is the dose calculated using the standard table (reference) and *D*_mod_ is the dose calculated using the modified table.

## Results

### Composition analysis

Table 2 shows a comparison between the elemental composition of BDP and that of the brain, as reported in ICRP 110; the MD was consistent between the two at 1.05 g/cm^3^. For the major elements, the hydrogen (H) content was 10.36% in BDP and 10.7% in ICRP 110. The carbon (C) content was 73.26% in BDP, compared to 14.3% in ICRP 110, while the oxygen (O) content was 12.52% in BDP and 71.3% in ICRP 110. The nitrogen (N) content was 1.99% in BDP and 2.3% in ICRP 110. Notably, significant differences were observed in the carbon-to-oxygen ratios. Furthermore, the EAN calculation yielded a value of 6.6, whereas the value for real brain tissue data from ICRP 110 was 7.4.

**Table 2:** Comparison of brain density plug composition with ICRP 110 Reference Data

	MD [g/cm^3^]	${w}_{H}$	${w}_{B}$	${w}_{C}$	${w}_{N}$	${w}_{O}$	${w}_{Na}$	${w}_{Mg}$	${w}_{Al}$	${w}_{Si}$
Component analysis	1.05	10.36	1.08	73.26	1.99	12.52	0.06	0.05	< 0.01	0.01
ICRP110	1.05	10.70	-	14.30	2.30	71.30	0.20	–	–	–
		${w}_{P}$	${w}_{S}$	${w}_{Cl}$	${w}_{K}$	${w}_{Ca}$	${w}_{Fe}$			
Component analysis		< 0.01	< 0.01	0.64	< 0.01	0.01	< 0.01			
ICRP110		0.40	0.20	0.30	0.30	–	–			

All values except mass density (MD) are given as percentages by weight. “-” indicates data not available in the ICRP 110 report. *MD* mass density, *ICRP* international commission on radiological protection

### Comparison of BDP and actual brain tissue

**Fig. 1:**
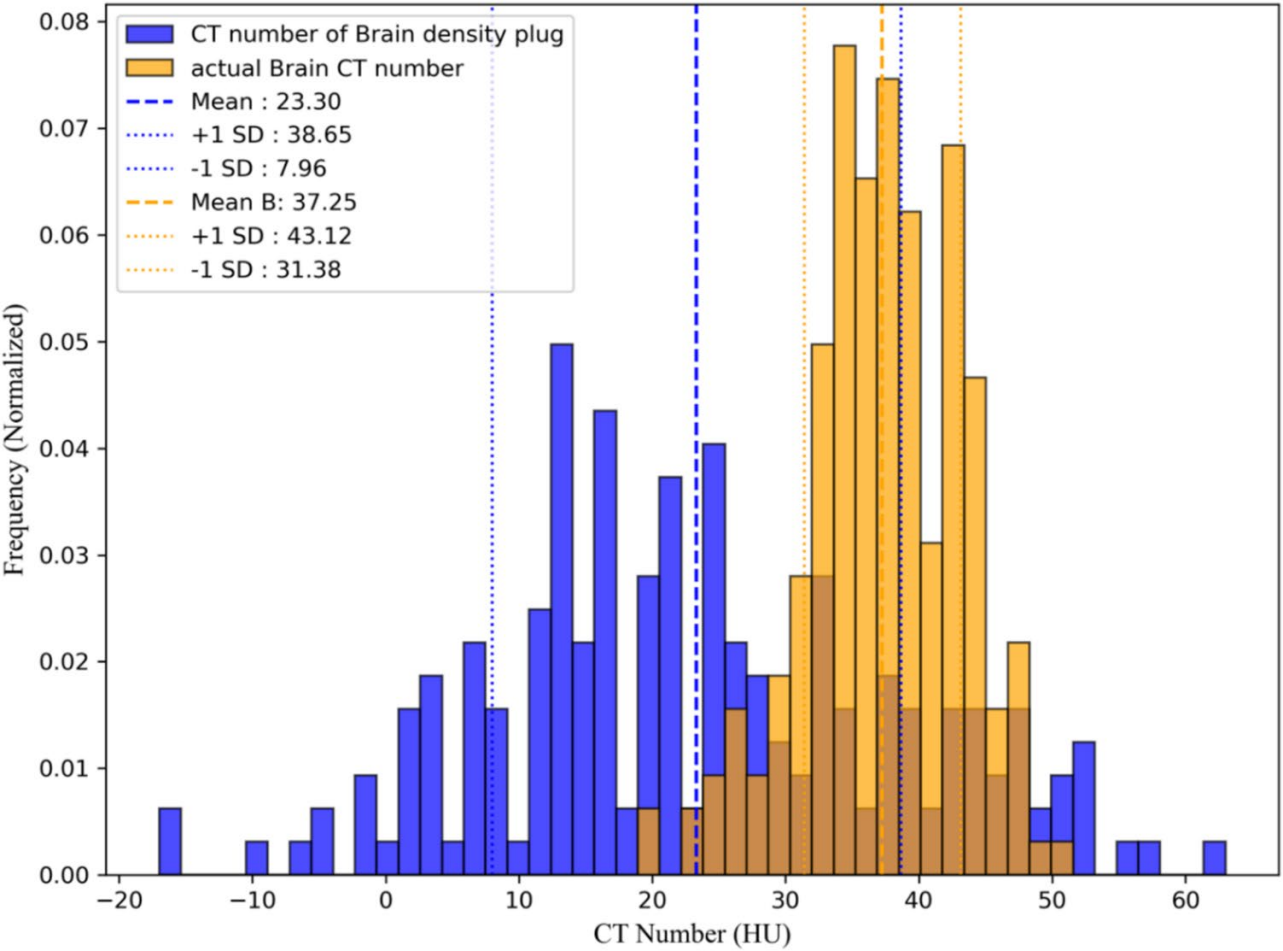
Histogram comparing CT numbers of BDP and actual brain tissue

**Fig. 2:**
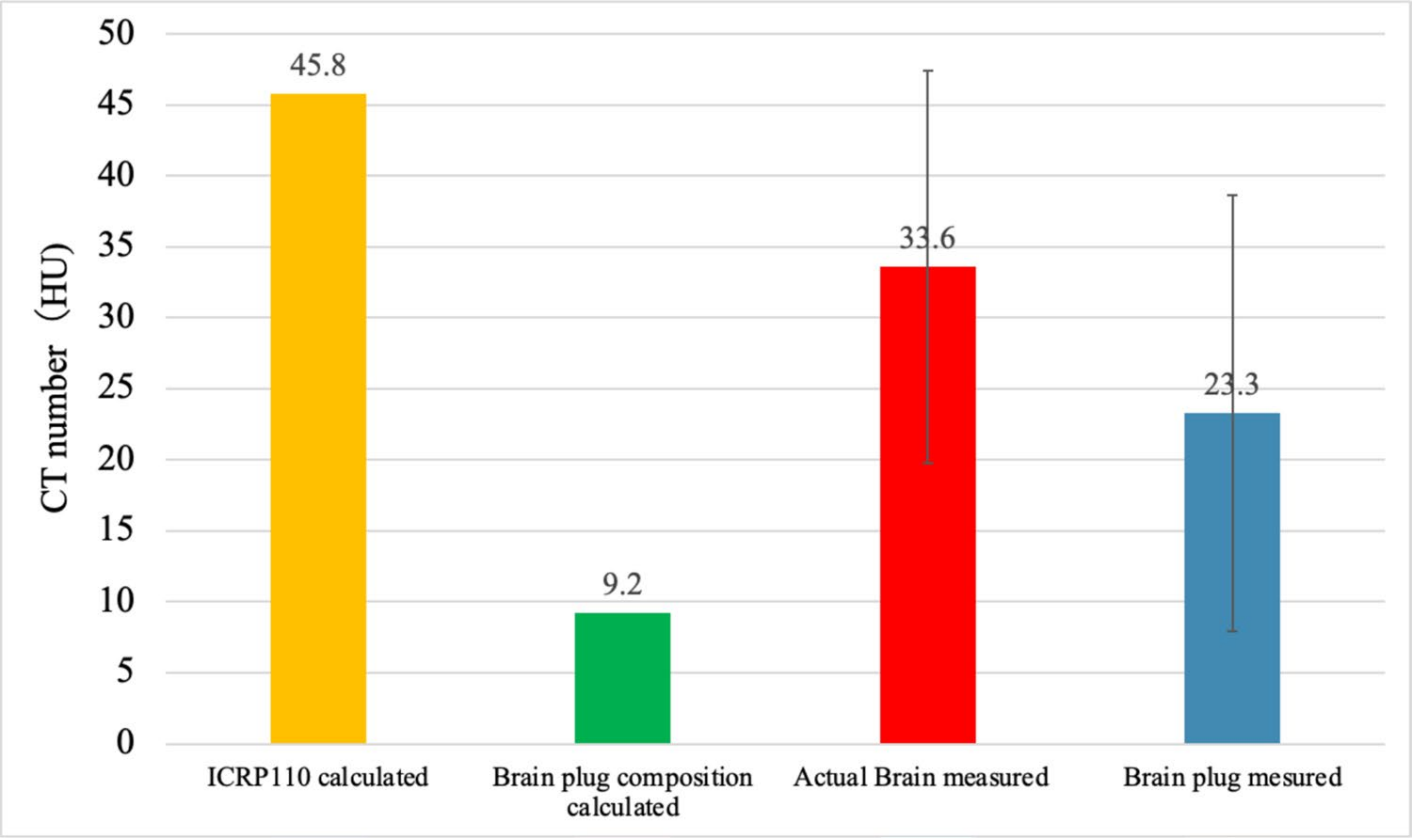
Comparison of calculated and measured CT numbers between BDP and actual brain tissue. The graph shows calculated and measured CT numbers (in Hounsfield units, HU) for different datasets: ICRP110 calculated (CT numbers based on actual brain data from ICRP110), Brain plug composition calculated (CT numbers derived from elemental composition analysis of the BDP), Actual brain measured (mean CT number measured from actual brain samples, *n* = 10), and Brain plug measured (CT numbers measured from the BDP). Error bars represent one standard deviation, indicating the variability in the measurements

Figure 1 shows a histogram comparing the CT numbers of individual pixels in the BDP and actual brain tissue. The CT numbers for all pixels within the ROI were recorded and the mean and SD were calculated to evaluate the variability. This figure represents a single example of actual brain tissue and the BDP, with ROIs of the same size used for both materials to ensure a fair comparison. The analysis indicated that the mean CT number of the BDP was 23.3 HU, with the majority of the values distributed within the range of 10–30 HU. The SD is approximately 15 HU, indicating a high degree of variability. Conversely, the mean CT number of actual brain tissue was 37.3 HU, with most values concentrated within the narrower range of 30–45 HU and an SD of 5.9 HU. The CT numbers of BDP exhibited a broader distribution (-10–60) to, ranging from (20–50 HU), unlike the uniform CT number distribution observed in actual brain tissue, indicative of a more homogeneous structure Fig. 2.

### Stoichiometric CT number calibration model

The mean CT numbers ± SD for the Tough lung and Tough bone plugs were –638.3 HU and 733.2 HU, respectively. CT numbers were used to construct the SCC model. The parameters *k*₁, *k*₂, and* α*, obtained through the least squares method for the SCC model, were 1.6 × 10⁻^3^, 2.7 × 10⁻^5^, and 0.98, respectively. Using this model, the calculated theoretical CT numbers were 9.2 HU for the Brain SR-2 plug and 45.8 HU for the actual brain data from ICRP 110.

### CT Number of actual brain tissue

Table 3 presents the CT numbers of the actual whole-brain tissue obtained from ten patient datasets using Eclipse version 16.1. The mean number for the actual whole-brain tissue was 33.6 HU ± 13.8 HU.

**Table 3:** Mean and standard deviation (SD) of CT numbers of actual brain tissue

Patient Case	mean (HU)	SD (HU)
1	33.8	16.1
2	33.9	13.0
3	31.5	13.9
4	34.7	14.2
5	36.8	13.1
6	31.9	12.9
7	33.8	13.0
8	34.4	13.4
9	34.7	14.2
10	30.6	14.3
Overall	33.6	13.8

Overall values are presented as mean values

### Comparison of calculated and measured CT numbers between actual brain and BDP

A comparison of the CT numbers between the actual brain tissue and BDP revealed significant discrepancies between the calculated and measured values. The CT number calculated using ICRP Publication 110 brain data was 45.8 HU, the highest among all datasets. In contrast, the CT number calculated based on the elemental composition of BDP was 9.2 HU, significantly lower than the other values. For the measured data, the mean CT number of the actual brain tissue was 37.25 HU, with minimal variability, as indicated by the narrow error bars. In comparison, the measured CT number for BDP was 23.3 HU, notably higher than the calculated value of 9.2 HU. Additionally, the calculated CT number of 45.8 HU for the ICRP110 brain data differed from the measured value of 33.6 HU for actual brain tissue, highlighting a gap between computational and measured results.

### Impact of CT-RED/MD table modifications on dose calculations in whole brain irradiation

Table 4 presents a comparison of the dose differences when applying different CT-RED/MD tables with the AAA and AcurosXB dose calculation algorithms. Dose differences were evaluated using Eq. (2) for both the modified table excluding the BDP and the modified table incorporating the ICRP Publication 110 brain data. For both algorithms, the dose differences associated with the modified CT-RED/MD tables were generally small, with most cases falling within ± 0.2%. Notably, the mean dose difference at D_50%_ remained at 0.1% for both algorithms.

**Table 4:** Comparison of dose differences using different CT-RED/MD tables and dose calculation algorithms in whole brain irradiation

Algorithm	Comparison	Case	D_98%_	D_50%_	D_2%_	D_reference_
AAA	Modified (BDP Excluded)	1	− 0.2%	− 0.2%	− 0.1%	0.3%
		2	0.2%	0.2%	0.1%	0.3%
		3	0.6%	0.2%	0.2%	0.3%
		Mean	0.2%	0.1%	0.0%	0.3%
		SD	0.3%	0.2%	0.1%	0.0%
AAA	Modified (ICRP110 Brain)	1	-0.1%	-0.1%	-0.1%	0.2%
		2	0.1%	0.1%	0.1%	0.1%
		3	0.6%	0.1%	0.6%	0.2%
		Mean	0.2%	0.1%	0.2%	0.2%
		SD	0.3%	0.1%	0.3%	0.0%
AcurosXB	Modified (BDP Excluded)	1	0.0%	0.1%	0.1%	0.1%
		2	0.1%	0.1%	0.1%	0.1%
		3	0.4%	0.1%	0.1%	0.1%
		Mean	0.2%	0.1%	0.1%	0.1%
		SD	0.2%	0.0%	0.0%	0.0%
AcurosXB	Modified (ICRP110 Brain)	1	-0.1%	-0.1%	0.0%	0.1%
		2	0.1%	0.1%	0.0%	0.2%
		3	0.6%	0.1%	0.1%	0.1%
		Mean	0.2%	0.1%	0.0%	0.2%
		SD	0.3%	0.1%	0.1%	0.0%

*AAA* anisotropic analytical algorithm, *D₉₈*_*%*_*, D₅₀*_*%*_*, D₂*_*%*_ dose received by 98%, 50%, and 2% of the target volume, respectively, *D*_*refrence*_ defined here as the prescribed dose covering the specified target volume (e.g., 50% of the PTV), modified (BDP Excluded); Comparison between CT-RED/MD table without BDP and original table. Modified (ICRP 110 Brain); Comparison between CT-RED/MD table using ICRP 110 brain data and the original table; *SD* standard deviation, *BDP* brain density plug

When using the modified table, excluding BDP, the mean dose differences for the AAA were 0.2%, 0.1%, 0.0% and 0.3% for D_98%_, D_50%_, D_2%_, and D_reference_ respectively. Similarly, the mean dose differences for Acuros XB were 0.2%, 0.1%, 0.1% and 0.1% respectively. In contrast, when utilizing the modified table based on the ICRP Publication 110 brain data, the mean dose differences for AAA were 0.2%, 0.1%, 0.2% and 0.2% whereas those for Acuros XB were 0.2%, 0.1%, 0.0% and 0.2% respectively. The largest dose difference of 0.6% at D_98%_ was observed in Case 3 for both algorithms. The SD across all conditions was less than 0.3%, reflecting minimal variability in the calculation Table 5.

**Table 5:** Comparison of dose differences using different CT-RED/MD tables and dose calculation algorithms in brain SRS/SRT

Algorithm	Comparison	Case	D_98%_	D_50%_	D_2%_	D_reference_
AAA	Modified (BDP Excluded)	1	0.1%	0.1%	0.0%	0.1%
		2	0.3%	0.3%	0.3%	0.3%
		3	0.2%	0.2%	0.2%	0.2%
		Mean	0.2%	0.2%	0.2%	0.2%
		SD	0.1%	0.1%	0.1%	0.1%
AAA	Modified (ICRP110 Brain)	1	0.0%	0.0%	0.0%	0.1%
		2	0.2%	0.2%	0.2%	0.2%
		3	0.1%	0.1%	0.1%	0.1%
		Mean	0.1%	0.1%	0.1%	0.1%
		SD	0.0%	0.1%	0.1%	0.0%
AcurosXB	Modified (BDP Excluded)	1	0.0%	0.0%	0.0%	0.1%
		2	0.1%	0.2%	-0.2%	0.2%
		3	0.1%	0.1%	0.1%	0.0%
		Mean	0.1%	0.1%	0.0%	0.1%
		SD	0.0%	0.1%	0.1%	0.1%
AcurosXB	Modified (ICRP110 Brain)	1	0.4%	0.1%	0.0%	0.1%
		2	0.2%	0.3%	− 0.1%	0.3%
		3	0.2%	0.2%	0.2%	0.0%
		Mean	0.3%	0.2%	0.0%	0.1%
		SD	0.1%	0.1%	0.1%	0.1%

*AAA* anisotropic analytical algorithm, *D₉₈*_*%*_*, D₅₀*_*%*_*, D₂*_*%*_ dose received by 98%, 50%, and 2% of the target volume, respectively, *D*_*refrence*_ defined here as the prescribed dose covering the specified target volume (e.g., 95% of the PTV), modified (BDP Excluded); Comparison between CT-RED/MD table without BDP and original table. Modified (ICRP 110 Brain); Comparison between CT-RED/MD table using ICRP 110 brain data and the original table; *SD* standard deviation, *BDP* brain density plug

### Impact of CT-RED/MD table modifications on dose calculations in brain SRS/SRT

When using the modified table, excluding BDP, the mean dose differences for AAA in SRS/SRT cases were 0.2%, 0.1%, 0.0%, and 0.2% for D_98%_, D_50%_, D_2%_, and D_reference_, respectively. Similarly, the mean dose differences for Acuros XB were 0.1%, 0.1%, 0.0%, and 0.1%, respectively. In contrast, when utilizing the modified table based on the ICRP Publication 110 brain data, the mean dose differences for AAA in SRS/SRT cases were 0.1%, 0.1%, 0.1%, and 0.1%, whereas those for Acuros XB were 0.3%, 0.2%, 0.0%, and 0.1%, respectively. The largest dose difference of 0.4% at D_98%_ was observed in Case 1 for Acuros XB. The SD across all conditions remained below 0.2%, indicating minimal variability in the dose calculations for SRS/SRT treatments.

## Discussion

In this study, we found compositional and CT number differences between commercially available BDP and actual human brain tissue and assessed their impact on dose calculation accuracy. Composition analysis revealed significant discrepancies in the elemental composition between BDP and actual brain tissue, as described in ICRP Publication 110 data. Notably, BDP exhibited a substantially higher carbon content (carbon: 14.3%, oxygen: 73.26%) and lower oxygen content (12.52%) than the inverse ratio in the actual brain tissue (oxygen: 71.30%). Consequently, the EANs differed by approximately one unit, with values of 6.6 for the BDP and 7.4 for actual brain tissue in ICRP data.

CT number comparisons also revealed notable discrepancies between the calculated and measured values for both the BDP and actual brain tissue. Using the SCC model, the theoretical CT number was calculated as 9.2 HU for the BDP and 45.8 HU for actual brain tissue, while measured mean CT numbers were approximately 23.3 HU for the BDP and 37.3 HU for brain tissue. These results indicate that despite being labeled as ‘brain-equivalent tissue’, the BDP does not fully replicate the attenuation properties of actual brain tissue. This discrepancy underscores the multifactorial nature of CT numbers, which are influenced by elemental composition and factors such as water content, microstructure, and scanning conditions [18]. Furthermore, the standard model derived from ICRP Publication 110 data may not fully reflect the actual clinical conditions, and inhomogeneities within the BDP itself likely contributed to the differences observed between the theoretical and measured CT numbers. Another possible explanation for the larger variance observed in BDP is related to its manufacturing process. Whereas actual brain tissue contains a high proportion of water, which helps maintain relatively uniform attenuation properties, artificial phantoms are more susceptible to small voids or inconsistencies arising during production. Consequently, these imperfections can lead to greater variations in CT numbers, thus diminishing the overall homogeneity of BDP when compared with actual brain tissue.

Despite compositional and CT number discrepancies, modifications to the CT-RED/MD tables resulted in dose distribution changes within clinically acceptable limits. The maximum deviation of dose-volume indices was approximately 0.6%, with most cases exhibiting deviations within ± 0.2%, thereby satisfying the 1–2% accuracy standard recommended by the American Association of Physicists in Medicine Task Group 85 [2]. Notably, D_98%_, which reflects low-dose regions, demonstrated greater sensitivity to compositional and density variations than D_50%_ and D_2%_, reaching a maximum difference of 0.6%. The consistency between the AAA and AcurosXB algorithms suggests that these dose differences are primarily attributable to modifications to the CT-RED/MD tables. We analyzed the results for both SRS/SRT and WBI. In WBI, the dose distribution is relatively uniform across the entire brain, resulting in minimal impact from modifications to the CT-RED/MD table. On the other hand, SRS/SRT, which involves steep dose gradients, was expected to be more sensitive to density variations. In comparison with WBI, SRS/SRT typically involves steeper dose gradients and is therefore theoretically more sensitive to density variations. However, in this study, the lesions were relatively small and did not introduce sufficient tissue heterogeneity to magnify the effects of modifying the CT-RED/MD table. As a result, even for highly conformal treatments targeting small volumes, the maximum variation in DVH indices was ± 0.4%, with most cases exhibiting deviations within ± 0.2%, which can be deemed clinically negligible. These findings suggest that, under typical clinical conditions and within standard calibration ranges, minor adjustments to the CT-RED/MD table for brain tissue are unlikely to produce substantial changes in DVH parameters. However, our results showed that even in SRS/SRT, changes in dose–volume indices remained within a maximum deviation of 0.4%, with most cases exhibiting variations within ± 0.2%.

These findings indicate that modifications to the CT-RED/MD table have only a limited impact on dose distributions for both WBI and SRS/SRT. Even in high-precision irradiation techniques such as SRS/SRT, the effect of CT-RED/MD table modifications on dose calculation accuracy remains within clinically acceptable limits. The analytical approach for AAA may be more sensitive to density variations, whereas AcurosXB appears to exhibit a lower sensitivity. Furthermore, the presence of structures with significantly different CT numbers (e.g., bones or ventricles) within the brain may have influenced the calculated dose distribution [18]. In addition, physical material Table in AcurosXB_13.5 assigns densities based on CT-RED/MD tables, and the range of densities corresponding to brain tissue includes values for muscle, skeletal tissue (ICRP, 1975), and cartilage (ICRP, 1975). Even if the CT values do not perfectly represent the brain tissue, AcurosXB recognizes them as the same material for calculation purposes if they fall within a defined range. This property may reduce the sensitivity of dose distribution to CT number inaccuracies.

A limitation of this study is that it focused on a specific RTPS (Eclipse) and particular dose calculation algorithms (AAA and AcurosXB). Further evaluations with alternative RTPSs and dose calculation algorithms, as well as analyses of the dose calculation results for non-brain tissues with mass densities similar to those of the BDP region (1.05 g/cm^3^), are required to confirm the generalizability of these findings. Monte Carlo methods are expected to produce results comparable to those of AcurosXB when the composition and mass density of the materials assigned based on the CT number to density table to each voxel are similar.

In summary, these results indicate that despite substantial compositional and CT number differences between the BDP and actual brain tissue, the current CT-RED/MD tables and BDP provide clinically acceptable dose calculation accuracy for RTPSs. However, the minor discrepancies observed in low-dose metrics (e.g., D_98%_) and the necessity for validation across different RTPSs highlight the importance of further refinement of the CT-RED/MD tables and additional research into their broader clinical implications.

## Conclusion

This study demonstrated that despite clear compositional and CT number differences between commercial BDP and actual brain tissue, the impact on dose calculation accuracy remained within clinically acceptable limits, with a maximum deviation of approximately 0.6% for dose–volume indices. However, minor effects were observed in low-dose regions (D_98%_), highlighting the sensitivity to density and compositional variations in dose calculation and emphasizing the need for further evaluation, including studies using alternative RTPS. These findings confirm the utility of standard tissue-equivalent plugs and suggest future improvements in accuracy and broader applicability will require refined CT-RED/MD tables and further research to reflect actual clinical conditions more accurately.

## Data Availability

The DICOM datasets generated and/or analyzed during the current study are not publicly available due to patient confidentiality and institutional policies. However, other datasets and supporting materials used in the analysis are available from the corresponding author upon reasonable request.
